# Oligosaccharide attenuates aging‐related liver dysfunction by activating Nrf2 antioxidant signaling

**DOI:** 10.1002/fsn3.1681

**Published:** 2020-06-05

**Authors:** Yueming Wang, Yanlei Xiong, Aiping Zhang, Nannan Zhao, Jiashen Zhang, Dongmei Zhao, Zhenhai Yu, Ning Xu, Yancun Yin, Xiying Luan, Yanlian Xiong

**Affiliations:** ^1^ Department of Anatomy School of Basic Medicine Binzhou Medical University Yantai China; ^2^ Department of Pathology Xuanwu Hospital Capital Medical University Beijing China; ^3^ Department of Immunology School of Basic Medicine Binzhou Medical University Yantai China

**Keywords:** anti‐aging, chitosan oligosaccharide, D‐galactose, liver, Nrf2 pathway

## Abstract

Chitosan oligosaccharide (COS) is the depolymerized product of chitosan possessing various biological activities and protective effects against inflammation and oxidative injury. The aim of the present study was to investigate the antioxidant effects of COS supplements on aging‐related liver dysfunction. We found that COS treatment significantly attenuated elevated liver function biomarkers and oxidative stress biomarkers and decreased antioxidative enzyme activities in liver tissues in D‐galactose (D‐gal)‐treated mice. Furthermore, COS treatment significantly upregulated the expression of Nrf2 and its downstream target genes HO‐1, NQO1, and CAT. Moreover, in vitro experiments showed that COS treatment played a vital role in protecting H_2_O_2_‐exposed L02 cells against oxidative stress by activating Nrf2 antioxidant signaling. These data indicate that COS could protect against D‐gal‐induced hepatic aging by activating Nrf2 antioxidant signaling, which may provide novel applications for the prevention and treatment of aging‐related hepatic dysfunction.

## Graphical abstract

Chitosan oligosaccharide (COS) treatment reduces inflammatory factors and oxidative stress biomarkers and attenuates aging‐related liver dysfunction. The underlying mechanisms are that COS upregulating the expression of Nrf2 and its downstream target genes HO‐1, NQO1, and CAT in liver tissue of aging mice.

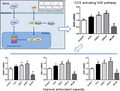

## 1 INTRODUCTION

Aging is a biological process characterized by progressive degeneration of physiological functions that results in an increase in the prevalence of morbidity and mortality. Hepatic cells are rich in mitochondria and prone to aging‐related injury and metabolic abnormalities due to mitochondrial reactive oxygen species (ROS) production (Luceri et al., 2018; Yang et al., 2019). Herein, effective antioxidants against ROS stress may be applied to protect against ROS‐induced liver dysfunction during the aging process.

NF‐E2‐related factor 2 (Nrf2) is a key transcription factor that controls many aspects of cell homoeostasis in response to oxidative and toxic insults (Wardyn, Ponsford, & Sanderson, 2015; Zhang, Yao, et al., 2019). The Nrf2 pathway regulates the expression of several antioxidant and detoxification enzymes including the catalytic subunits of glutamate–cysteine ligase (GCLC), heme oxygenase‐1 (HO‐1), and NAD(P)H quinone oxidoreductase 1 (NQO1) by binding to the antioxidant response element (ARE) in their promoter regions (Kubben et al., 2016). Previous studies have shown that Nrf2 is an essential regulator of longevity (Bruns et al., 2015). Current studies suggest that chitosan oligosaccharide (COS) activates numerous antioxidant genes and promotes Nrf2 translocation (Hyung, Ahn, Il Kim, Kim, & Je, 2016; Zhang, Ahmad, et al., 2019). However, whether COS can regulate the oxidative and antioxidant balance in aging cells by regulating the expression of Nrf2 pathway remains unclear.

Chitosan oligosaccharide is prepared from the degradation of chitosan and is a mixture of β‐1,4‐linked D‐glucosamine residue oligomers and abundant in insect cell walls and crustacean exoskeletons. Studies have shown that COS possesses a wide range of biological effects including anti‐inflammation, antioxidation, antitumor, anti‐Alzheimer's disease, antihypertension, and anti‐obesity (Azuma, Osaki, Minami, & Okamoto, 2015; Muanprasat & Chatsudthipong, 2017). Furthermore, there is also evidence showing that COS has an anti‐aging role via inhibition of cellular senescence and maintaining a favorable redox balance in mice triggered by D‐galactose (D‐gal) (Kong et al., 2018).

Although previous studies have already demonstrated that COS possesses hepatoprotective and renoprotective effects in D‐gal‐induced subacute aging mice to realize its anti‐aging activity (Kong et al., 2018), the effects of COS on aging‐related liver injury triggered by D‐gal and its potential molecular mechanism remain to be explored. Herein, we investigated the effects of COS on liver injury at two dose levels using a D‐gal‐induced animal aging model.

## 2 MATERIALS AND METHODS

### 2.1 Chemicals and reagents

D‐gal and ML385 were obtained from Sigma‐Aldrich Chemical Co. COS (average molecular weight <1,000, DD. 91.3%) was obtained from Dalian Glycobio Co., Ltd. A certain amount of COS was weighed accurately and dissolved in ultrapure water at room temperature to prepare two different concentrations: 15 and 60 mg/ml. Liver function biomarkers assay kits were obtained from BioVision (Cat. No: K553, E4320, and E4324, Inc.). The MTT cell assay kit, oxidative stress, and antioxidant biomarkers assay kits were obtained from Nanjing Jiancheng Bioengineering Institute: MTT (cat. no. G020‐1‐1), AGEs (cat. no. abx512406), 8‐OH‐dG (cat. no. SKT‐120‐480), ROS (cat. no. E004‐1‐1), GSH‐Px (cat. no. A005‐1‐2), SOD (cat. no. A001‐3‐2), and CAT (cat. no. A007‐1‐1). The following antibodies were used: GAPDH (#5174) and Nrf2 (#12721) (Cell Signaling Technology). All other chemicals and reagents used in the study were purchased from Sigma‐Aldrich.

### 2.2 Animals

Male C57BL/6 mice at 8 weeks of age were obtained from the animal center of Binzhou Medical University. Animals were fed according to the national standard rodent feed. All mice were kept in the condition that relative humidity is 45%–55%, room temperature is (22 ± 3°C), and the light time is 8:00–20:00. The experiments were performed in accordance with protocols approved by the Animal Ethics Committee of Binzhou Medical University (Permit Number: 2018‐0001). Animals were treated in accordance with Guide for the Care and Use of Laboratory Animals (8th edition, National Academies Press).

After adaptation for 1 week, 50 mice were randomly divided into four groups as follows: (a) control group: Mice were treated with saline (20 ml kg^−1^ day^−1^) as a vehicle for 7 weeks; (b) D‐gal group: Mice were treated with D‐gal (200 mg kg^−1^ day^−1^) for 7 consecutive weeks; (c) COS group: 20 mice were randomly divided further into two subgroups (COS‐L and COS‐H groups) by COS‐treated doses. After D‐gal (200 mg kg^−1^ day^−1^) intraperitoneal injection in mice for 1 hr, mice in COS group were treated with COS (COS‐L group: 300 mg kg^−1^ day^−1^ and COS‐H group: 1,200 mg kg^−1^ day^−1^) by gavage for 7 weeks. (d) VE group: Mice were treated with VE by gavage (200 mg kg^−1^ day^−1^) for 7 consecutive weeks as a positive control according to recent reports (Kong et al., 2018).

### 2.3 Detection of liver index

The mice and their livers were weighed 30 min after the last administration, and the liver index was calculated according to the following equation: Liver index = weight liver wet weight/body weight × 100%.

### 2.4 Detection of liver function biomarkers

Serum were separated for the assess of aspartate aminotransferase (AST) and alanine aminotransferase (ALT) levels using commercially available colorimetric assay kits. Serum total bilirubin (TBIL) and direct bilirubin (DBIL) were assessed using Bilirubin (Total and Direct) colorimetric assay Kit.

### 2.5 Detection of liver malondialdehyde (MDA)

Malondialdehyde was quantified as thiobarbituric acid reactive substances (TBARS). Briefly, the weighed samples were homogenized in 1 ml 5% trichloroacetic acid. The samples were centrifuged (10,000 *g*), and 250 ml of the supernatant was reacted with the same volume of 20 mM thiobarbituric acid for 35 min at 95°C, followed by 10 min at 4°C. Sample fluorescence was read using a spectrophotometric plate reader (Victor3 1420‐050; Perkin Elmer) with an excitation wavelength of 515 nm and an emission wavelength of 553 nm.

### 2.6 Detection of 8‐Hydroxy‐2′‐deoxyguanosine (8‐OH‐dG)

The liver pellets were resuspended, and the DNA was isolated using the method recommended by ESCODD. The 8‐OH‐dG in the DNA was detected using an ESA Coulochem II electrochemical detector in line with a UV detector as previously described (Lodovici et al., 2007).

### 2.7 Detection of advanced glycation end‐products (AGEs)

The supernatant was collected as described above, and the levels of AGEs in the liver in each group were measured by ELISA kits according to the manufacturer's protocol.

### 2.8 Detection of proinflammatory cytokines

The liver tissues were homogenized in PBS, centrifuged at 10,000*g* for 15 min, and collected supernatants for the measurement of cytokines, including monocyte chemoattractant protein‐1(MCP1), tumor necrosis factor alpha (TNF‐α), and IL‐6 (interleukin‐6) by ELISA kits, and the detection methods were performed according to the manufactures' instruction.

### 2.9 Detection of liver antioxidant markers biomarkers

An equal amount of liver was homogenized, and the supernatants were used for the detection. The supernatant was collected after centrifugation at 12,000 rpm and 4°C for 15 min. GSH‐Px, CAT, and SOD activity were detected by colorimetric analysis according to the manufacturer's protocol (Xiao et al., 2018).

### 2.10 Histopathological analysis

Liver tissues were fixed in 4% paraformaldehyde for 12–24 hr, dehydrated in absolute ethanol, transparentized in dimethylbenzene, and embedded in paraffin. Sections of 4 µm were cut, mounted on glass slides, deparaffinized, dehydrated with gradient ethanol, and routinely stained with hematoxylin–eosin (HE), and sealed with optical resin. The stained sections were observed by light microscopy (Nikon).

### 2.11 Western blot analysis

Total cell extracts were prepared in 1 × sodium dodecyl sulfate–polyacrylamide gel electrophoresis (SDS‐PAGE) sample loading buffer. Cell fractions were extracted with nuclear and cytoplasm protein extraction kit (Wanleibio). Cell proteins were resolved by SDS‐PAGE and transferred to a polyvinylidene difluoride membrane. The membranes were incubated with primary antibodies overnight at 4°C and appropriate HRP‐secondary antibodies for 1 hr at room temperature. Detection was performed using a Thermo Scientific Pierce enhanced chemiluminescence Western blotting substrate (Thermo Scientific) (Xiong et al., 2016).

### 2.12 Cell culture and group assignment

L02 cells were obtained from the Type Culture Collection of the Chinese Academy of Sciences. Cells were divided into five groups and were cultured with corresponding medium supplied with different reagents. The five groups included: (a) control group; (b) H_2_O_2_ group (cells were incubated with 200 μM H_2_O_2_ for 12 hr); (c) COS‐L group (cells were pretreated with 100 μg/ml COS for 12 hr; then, cells were treated with 200 μM H_2_O_2_ in combination for 24 hr); (d) COS‐H group (cells were pretreated with 100 μg/ml COS for 12 hr; then, cells were treated with 200 μM H_2_O_2_ in combination for 24 hr); and (e) ML385 group (ML385 was used to inhibit the expression of Nrf2 (Xu et al., 2019). Cells were pretreated with 300 μg/ml COS and 10 μM ML385 for 12 hr; then, cells were treated with 200 μM H_2_O_2_ in combination for 24 hr.

### 2.13 RNA extraction and quantitative real‐time PCR

Total RNA was isolated using TRIzol reagent as manufacturer's instructions. We performed real‐time PCR assay by using SYBR green dye on Step One sequence detection system (ABI). Using β‐actin as internal control, we calculated relative abundance of genes using 2^−∆∆CT^ formula. Primers attached in the Table 1.

**TABLE 1:** Sequences used for RT‐qPCR

Name	Accession No	Primer sequences (Forward/Reverse primer)	Product length (bp)
Nrf2	NM_001114671.1	GACAAACCGCCTCAACTCAG GTCTCCACGTCGTAGCGTTC	183
NQO1	NM_001159613.1	GATCATACTGGCCCACTCCG GAGCAGTCTCGGCAGGATAC	200
HO‐1	NM_001004027.1	CAAGCAGAAAATCCTCGAAG GCTGAGTGTCAGGACCCATC	241
CAT	XM_021081498.1	AGCTTTGCCCTTGCACAAAC ACATCCTGAACAAGAAGGGGC	119
β‐actin	XM_003124280.4	CACGCCATCCTGCGTCTGGA AGCACCGTGTTGGCGTAGAG	380

### 2.14 Detection of ROS

The intracellular ROS levels were measured using the ROS assay kit according to the manufacturer's instructions. Briefly, The L02 cells were plated in a 96‐well plate (5 × 10^4^ cells/well). After the COS and H_2_O_2_ treatment, the cells were washed with Hanks balanced salt solution (HBSS) and incubated with 500 μM of the luminol derivative L02 in HBSS at 37°C for 15 min. ROS‐induced chemiluminescence was determined every 10 min for a total of 60 min using a Microplate Luminometer (Tropix).

### 2.15 Cell viability assay

Cell viability was determined by MTT assay. After the COS and H_2_O_2_ treatment, MTT was added and incubated for 4 hr at 37°C. Subsequently, the plate was centrifuged at a speed of 800 *g* for 5 min and the supernatant was discarded. Then, the formazan crystals formed in each well were dissolved using 100 μl DMSO and the absorbance was measured at a wavelength of 540 nm. The relative cell viability was calculated by comparison with the absorbance of untreated control group.

### 2.16 Statistical analysis

Results are expressed as mean ± *SEM* of three independent experiments in triplicate. Data were conducted by one‐way analysis of variance (ANOVA) followed by Tukey's post hoc test. Significance was defined as *p* < .05.

## 3 RESULTS

### 3.1 COS alleviated D‐gal triggered liver dysfunction in mice

**FIGURE 1:**
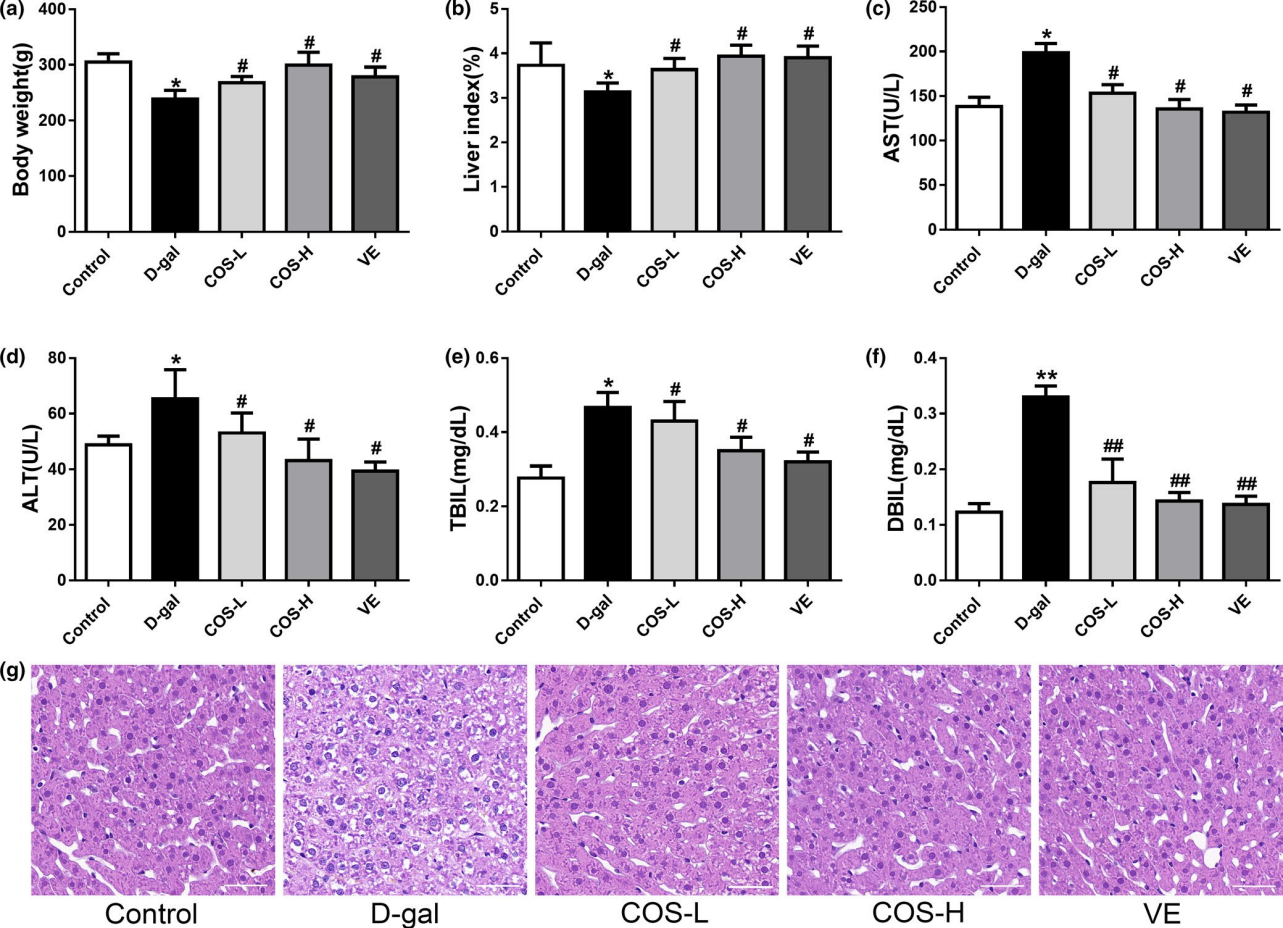
The effect of COS on body weight and liver function biomarkers of D‐gal‐treated mice. (a) Effect of COS on the body weight. (b) Effect of COS on the liver index. Effect of COS on serum levels of AST (c), ALT (d), TBIL (e), and DBIL (f). (g) Histological examination of liver sections stained with H&E (bar = 50 μm). *p < .05, compared with the control group; **p < .01, compared with the control group; ^#^ *p* < .05, compared with the D‐gal model group; ^##^ *p* < .01, compared with the D‐gal model group

As shown in Figure 1a,b, administration of D‐gal significantly reduced the body weight and liver index compared with the control group (*p* < .05). At the same time, administration of VE or COS‐H could significantly increase the body weight and liver index compared with the D‐gal group (*p* < .05).

Serum liver function biomarkers levels of AST, ALT, TBIL, and DBIL were significantly increased in D‐gal group compared to the control group (*p* < .05) (Figure 1c–f). Treatment with COS or VE significantly restored liver function markers to normal levels compared with D‐gal group (*p* < .05).

Representative photomicrographs of the HE‐stained liver tissues are shown in Figure 1g. The control group exhibited a normal lobular architecture and radiating hepatic cords, whereas mice in D‐gal group contained hepatocytes that were disorganized and showed irregular morphology, distorted hepatic cords, vacuolation, and widespread signs of inflammatory infiltration. Treatment of COS significantly ameliorated pathohistological alterations in the liver of mice triggered by D‐gal in a dose‐dependent manner.

### 3.2 Effect of COS on liver oxidative stress biomarkers and inflammatory cytokines of D‐gal‐treated mice

**FIGURE 2:**
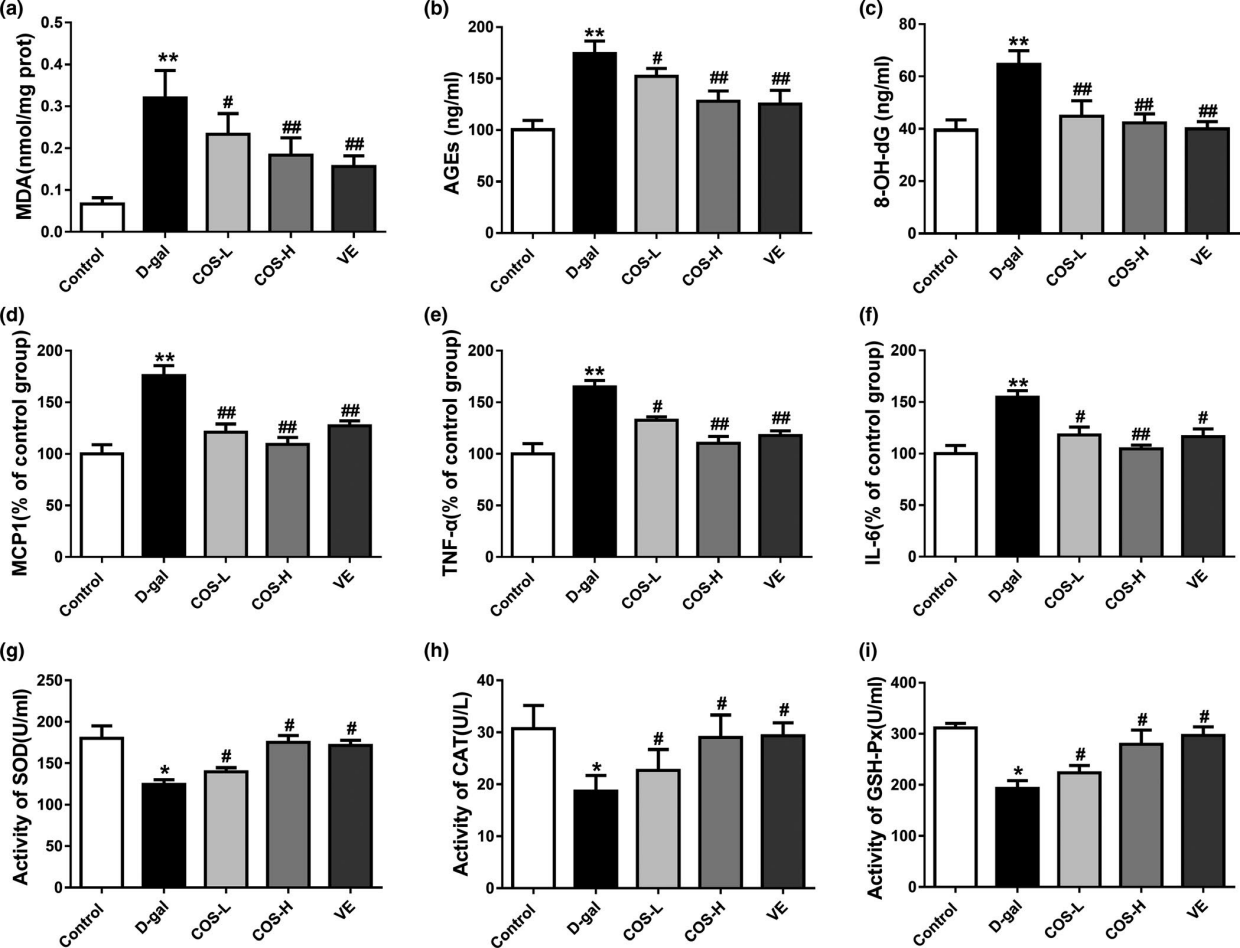
The effect of COS on liver proinflammatory cytokines, oxidative stress, and antioxidant markers of D‐gal‐treated mice. (a) Effect of COS on the MDA level of liver tissue. (b) Effect of COS on the AGEs level of liver tissue. (c) Effect of COS on the 8‐OH‐dG level of liver tissue. (d) Effect of COS on the MCP1 level of liver tissue. (e) Effect of COS on the TNF‐α level of liver tissue. (f) Effect of COS on the IL‐6 level of liver tissue. (g) Effect of COS on the SOD activity of liver tissue. (h) Effect of COS on the CAT activity of liver tissue. (i) Effect of COS on the GSH‐Px activity of liver tissue. *p < .05, compared with the control group; **p < .01, compared with the control group; ^#^ *p* < .05, compared with the D‐gal model group; ^##^ *p* < .01, compared with the D‐gal model group

There was a markedly increase in the oxidative stress biomarkers of MDA, AGEs, and 8‐OH‐dG levels in the D‐gal group (*p* < .01), while COS or VE treatment significantly decreased MDA, AGEs, and 8‐OH‐dG levels compared with D‐gal group (*p* < .05) (Figure 2a–c). In addition, the levels of MDA, AGEs, and 8‐OH‐dG levels decreased significantly by 42.8%, 26.6%, and 34.6% in COS‐H group compared with D‐gal group, respectively.

We further investigated the changes in expression of proinflammatory cytokines in 7 weeks continuous administration of COS. Levels of MCP1, TNF‐α, and IL‐6 significantly increased in the D‐gal group (*p* < .01), while COS or VE treatment significantly decreased MCP1, TNF‐α, and IL‐6 levels compared with D‐gal group (*p* < .05) (Figure 2d–f). In addition, the levels of MCP1, TNF‐α, and IL‐6 levels decreased significantly by 37.9%, 33.0%, and 32.3% in COS‐H group compared with D‐gal group, respectively.

### 3.3 Effect of COS on the liver antioxidant biomarkers of D‐gal‐treated mice

Liver antioxidant markers, namely SOD, CAT, and GSH‐Px activities, showed significant decline in D‐gal‐treated group compared with control group (*p* < .05), while COS or VE treatment significantly increased SOD, CAT, and GSH‐Px activities compared with D‐gal group (*p* < .05). The SOD, CAT, and GSH‐Px activities elevated significantly by 40.8%, 55.4% and 44.6% in COS‐H group compared with D‐gal group, respectively (Figure 2g–i).

### 3.4 Effect of COS on the expressions of Nrf2 pathway in the liver tissue of D‐gal‐treated mice

**FIGURE 3:**
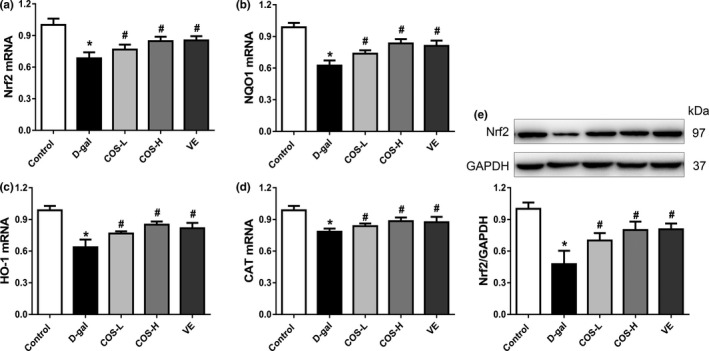
The effect of COS on the expressions of Nrf2 signaling pathway in the liver tissue of D‐gal‐treated mice. (a) The mRNA expressions of Nrf2. (b) The mRNA expressions of NQO1. (c) The mRNA expressions of HO‐1. (d) The mRNA expressions of CAT. (e) The protein expressions of Nrf2. *p < .05, compared with the control group; ^#^ *p* < .05, compared with the D‐gal group

We further investigated whether COS initiated the activation of Nrf2 pathways. As shown in Figure 3a,e, the protein and mRNA expression of Nrf2 were decreased significantly in liver tissue in D‐gal group in compared with control group (*p* < .05). Meanwhile, COS had managed to significantly increase in the mRNA and protein levels of Nrf2 in liver tissue in a dose‐dependent manner compared with D‐gal group (*p* < .05 for all) (Figure 3a,e). In addition, the mRNA expression levels of Nrf2 downstream target gene NQO1, HO‐1, and CAT decreased significantly in D‐gal‐treated group compared with control group (*p* < .05 for all) (Figure 3b–d), while COS or VE treatment significantly increased the mRNA expression levels of NQO1, HO‐1, and CAT compared with D‐gal group (*p* < .05 for all).

### 3.5 Effects of COS on viability and ROS levels of H_2_O_2_‐treated L02 cells

**FIGURE 4:**
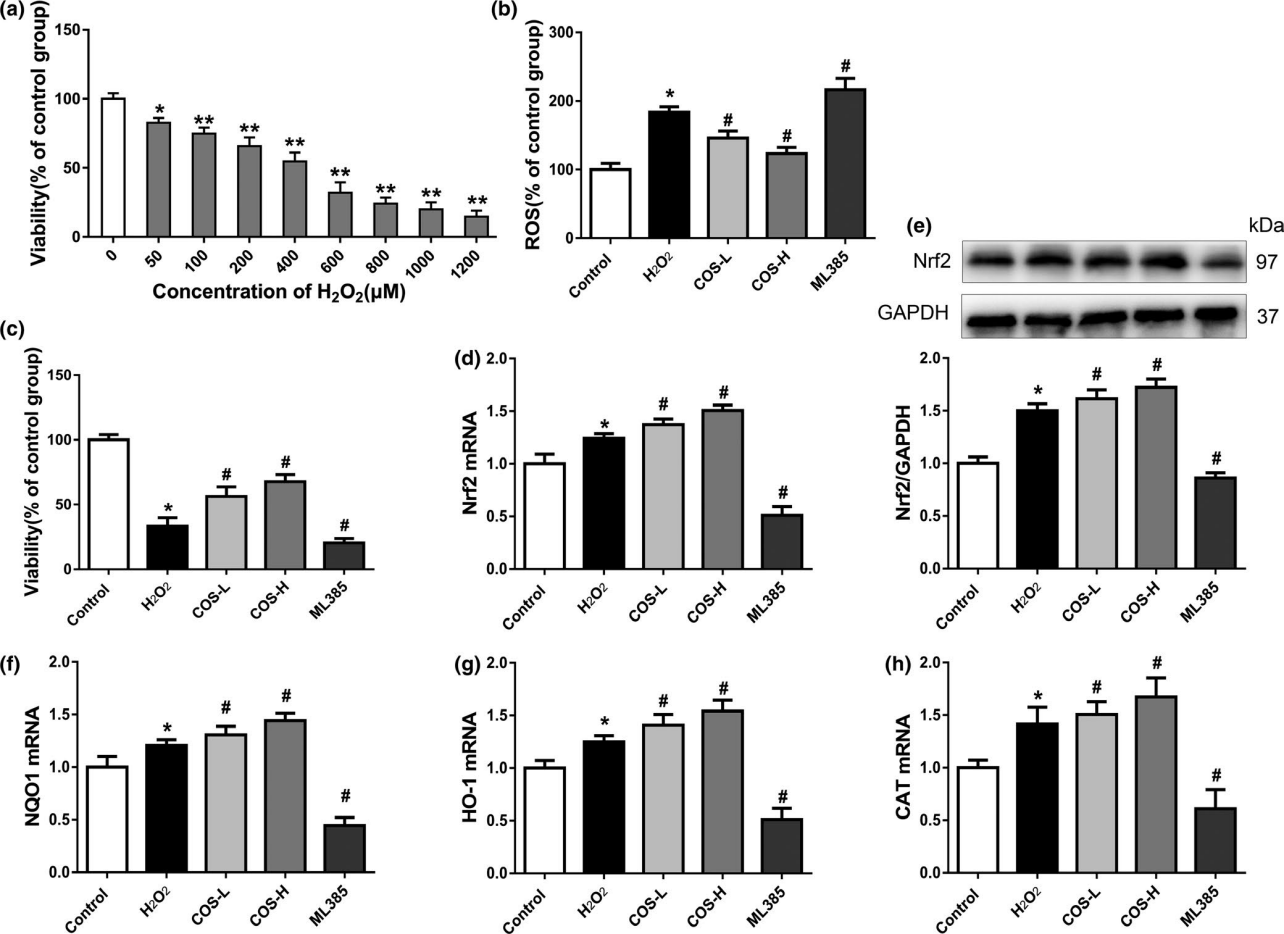
The effects of COS on the viability, ROS contents, and the expressions of Nrf2 pathway of H_2_O_2_‐treated L02 cells. (a) Effects of gradient concentration H_2_O_2_ treatment on the viability of L02 cells. (b) Effects of COS on the ROS contents of H_2_O_2_‐treated L02 cells. (c) Effects of COS on the viability of H_2_O_2_‐treated L02 cells. (d) Effects of COS on the mRNA expressions of Nrf2. (e) Effects of COS on the protein expressions of Nrf2. (f‐h) Effects of COS on the mRNA expressions of NQO1, HO‐1 and CAT. *p < .05, **p < .01, compared with the control group; ^#^ *p* < .05, compared with the H_2_O_2_ treatment group

We constructed an H_2_O_2_ oxidation model in vitro to simulate oxidative stress microenvironment in aging organisms and to explore the molecular mechanism by which COS alleviating dysfunction of liver cells. As shown in Figure 4a, with increasing concentrations of H_2_O_2_, cell viability decreased in a dose‐dependent manner. Then, treating the L02 cells with H_2_O_2_ at a final concentration of 200 μM was selected for subsequent experiments. As shown in Figure 4b,c, exposure to 200 μM H_2_O_2_ significantly decreased the viability and increased the ROS levels in L02 cells (*p* < .01). In addition, pretreatment with 100 or 300 μg/ml COS significantly increased the cell viability and reduced ROS levels in L02 cells compared with H_2_O_2_ group (*p* < .05).

### 3.6 Effect of COS on the expressions of Nrf2 pathway of H_2_O_2_‐treated L02 cells

The mRNA and protein levels of Nrf2 were increased significantly after incubated with 200 μM H_2_O_2_ for 12 hr. In addition, pretreated with 100 or 300 μg/ml COS can further promote the expression of Nrf2 mRNA and protein compared with H_2_O_2_ group (Figure 4d,e). In turn, COS treatment significantly increased the mRNA expression levels of NQO1, HO‐1, and CAT (*p* < .05 for all) (Figure 4f–h). Furthermore, the intervention of Nrf2 inhibitor ML385 induces an significant downregulation of Nrf2 and downstream gene expression, accompanied by a significant increase in ROS levels and significant decrease in cell viability (*p* < .05 for all) (Figure 4b–h). This result demonstrates that COS treatment leads to the activation of Nrf2 pathway in H_2_O_2_‐treated L02 cells.

## 4 DISCUSSION

Oxidative damage has long been implicated in the aging process. Dietary antioxidant supplementation may be an effective treatment for the correction of impaired plasma membrane redox systems by reducing the incidence of aging‐related diseases in the elderly (Chen, Chen, & Zhou, 2018; Xiao et al., 2018; Xiong et al., 2017). In this study, D‐gal‐induced subacute aging in mice was chosen to investigate the possible anti‐aging effects of COS and explore the underlying mechanism. Our study demonstrates that COS treatment could induce Nrf2 pathway activation and subsequently upregulate the expression of the downstream target genes NQO1, HO‐1, and CAT. These antioxidant genes further play vital roles in scavenging free radicals in liver tissues of D‐gal‐treated mice. Additionally, in vitro experiments showed that COS treatment protected H_2_O_2_‐exposed L02 cells against oxidative stress by activating Nrf2 antioxidant signaling. These findings uncover some novel molecular events in COS anti‐aging biological effects.

Aging is associated with the gradual alteration of hepatic structure and function as well as various changes in liver cells including hepatic sinusoidal endothelial cells (Kim et al., 2016). In the present study, we observed a significant elevation in liver enzyme biomarkers, including ALT, AST, TBIL, and DBIL, in D‐gal‐treated mice compared to control group mice. Although it does not meet the clinical classification standard of drug‐induced liver injury (elevate 2–5 times), it still revealed that D‐gal induced dysfunction of liver metabolism. And it was in line with previous studies that D‐gal aging model could induce significant elevate in liver function biomarkers (Saleh, Mansour, Hashad, & Bakeer, 2019). Furthermore, COS treatment significantly relieved the abnormalities in serum liver functions in D‐gal‐treated mice in a dose‐dependent manner. Recently, Chen et al. (2018) found that upregulation of liver aminotransferases during liver aging induced by D‐gal is considered an early sensitive marker of liver injury. Additionally, the process of bilirubin catabolism is mainly dependent upon liver functions; therefore, high levels of bilirubin may reflect hepatocellular dysfunction (Boland, Dong, Bettencourt, Barrett‐Connor, & Loomba, 2014). Studies have previously demonstrated that oxidative stress is an important cause of oxidative injury and inflammation in liver tissues during the aging process (Liu et al., 2019; Saleh et al., 2019). D‐gal treatment induction of high fibrotic areas in the liver is a result of hepatic oxidation and inflammation, which consequently activate fibrogenic responses and promote liver fibrosis (Huang et al., 2013). D‐gal‐induced osmotic stress promotes deterioration of the antioxidant defense and the overproduction of reactive oxygen species in the aging process (Bo‐Htay, Palee, Apaijai, Chattipakorn, & Chattipakorn, 2018). MDA and 8‐OH‐dG are end‐products of ROS‐induced lipid peroxidation and DNA oxidation that are commonly used as oxidative stress biomarkers (Mizoue et al., 2007; Xiao et al., 2018). AGEs are nonenzymatic glycosylation reaction end‐products and considered to be associated with aging‐related inflammation and oxidation in liver tissue (Hollenbach, 2017). Our current study showed that continuous administration of COS significantly downregulated MDA, AGE, and 8‐OH‐dG contents, while significantly upregulating the activities of the antioxidative enzymes SOD, CAT, and GSH‐Px in liver tissues in D‐gal‐treated mice. SOD, CAT, and GSH‐Px are important enzymes that participate in the removal of ROS from the cellular environment (Kong et al., 2018). These results indicated that COS may play an anti‐aging role by enhancing the activity of endogenous antioxidative enzymes in oxidized cells and reducing the levels of peroxidation products.

Nrf2 is a transcription factor that regulates various antioxidation and detoxification enzymes (Ahmed, Luo, Namani, Wang, & Tang, 2017; Ambrozewicz et al., 2018; Zhang, Yao, et al., 2019). Nrf2 is normally bound to the reduced form of Keap1 and is inactive in the cytoplasm. However, under oxidative stress conditions, Nrf2 is released from oxidized Keap1 and translocated to the nucleus where it binds to the ARE in the promoter region of target genes, including HO‐1, NQO1, and CAT (Kwak, Wakabayashi, Greenlaw, Yamamoto, & Kensler, 2003; Lee, Calkins, Chan, Kan, & Johnson, 2003). Numerous studies have proven that the activity of the Nrf2 pathway is closely related to redox regulation in aging diseases (Han, Nan, Fan, Chen, & Zhang, 2019; Morroni et al., 2018; Vasconcelos, Dos Santos, Scavone, & Munhoz, 2019; Zhu et al., 2019). In the present study, we observed that D‐gal treatment downregulating the expression of Nrf2 and its downstream target genes HO‐1, NQO1, and CAT. In vitro studies demonstrated that COS treatment reduced ROS accumulation and increased the viability of H_2_O_2_‐exposed L02 cells by upregulating the expression of Nrf2 and its downstream target genes. Furthermore, intervention with the Nrf2 inhibitor ML385 induces an significant decrease in the expression of Nrf2 and its downstream target genes, accompanied by a significant increase in ROS levels and significant decrease in viability of H_2_O_2_‐exposed L02 cells. This reveals the therapeutic effects of COS on aging‐related oxidative stress injure of liver via activating the Nrf2 antioxidant pathway. These results are in agreement with previous results that COS prevents apoptosis and oxidative stress in mice and H9C2 cells by activating the Nrf2/ARE pathway (Zhang, Ahmad, et al., 2019).

## 5 CONCLUSIONS

In summary, our study demonstrated that COS provides protection against D‐gal‐induced inflammatory, oxidative stress, and liver dysfunction. Furthermore, COS initiates protective effects by activating Nrf2 and its downstream target genes. These findings provide novel applications of COS to prevent and treat aging‐related hepatic dysfunction.

## CONFLICT OF INTEREST

The authors have declared that there is no conflict of interest.

## ETHICAL APPROVAL

Animal experiments were performed in accordance with the National Guideline for Experimental Animal Welfare and with approval by the Animal Ethics Committee of Binzhou Medical University (Permit Number: 2018‐0001).
